# A note on $(p,q)$-Bernstein polynomials and their applications based on $(p,q)$-calculus

**DOI:** 10.1186/s13660-018-1673-3

**Published:** 2018-04-10

**Authors:** Erkan Agyuz, Mehmet Acikgoz

**Affiliations:** Department of Mathematics, Faculty of Arts and Science, University of Gaziantep, Gaziantep, Turkey

**Keywords:** 05A15, 05A30, 11B65, 65Q20, $(p,q)$-calculus, $(p,q)$-Bernstein polynomials, Generating function, Functional equations

## Abstract

Nowadays $(p,q)$-Bernstein polynomials have been studied in many different fields such as operator theory, CAGD, and number theory. In order to obtain the fundamental properties and results of Bernstein polynomials by using $(p,q)$-calculus, we give basic definitions and results related to $(p,q)$-calculus. The main purpose of this study is to investigate a generating function for $(p,q)$-Bernstein polynomials. By using an approach similar to that of Goldman et al. in (SIAM J. Discrete Math. 28(3):1009-1025, 2014), we derive some new identities, relations, and formulas for the $(p,q)$-Bernstein polynomials. Also, we give a plot generating function of $(p,q)$-Bernstein polynomials for some selected *p* and *q* values.

## Introduction

The generating functions are the key tool in analytic number theory for defining and obtaining properties to special polynomials such as Bernoulli, Euler, Hermite, and Genocchi polynomials. The idea behind the generating functions are that we can effectually transform problems about sequences into problems about functions. The generating functions have many advantages. For instance, we can apply various operations on generating functions such as scaling, addition, product, integration, and differentiation.

The Bernstein polynomials, which are called by the name of their creator [2], have many applications in mathematics, engineering, CAGD, and statistics. The reason for the emergence of Bernstein polynomials is the study of the existence of polynomials that are constantly approaching each function at $[ 0,1 ] $. Many useful and important studies have been carried out in approximation theory with the definition of Bernstein polynomials (see [3] and [4]). Phillips obtained Bernstein polynomials that depend on *q*-integers [5]. Oruc and Phillips defined *q*-Bernstein polynomials and Bezier curves and showed useful results for these polynomials and curves [6]. Lewanowicz and Wozny obtained some applications and found interesting formulae for Bezier representations and coefficients [7]. Khan and Lobiyal studied Lupaş type $(p,q)$-Bernstein operators and Lupaş type $(p,q)$-Bezier curves and surfaces [8]. Perez and Palacin proposed an estimation for a quantile function by using the Bernstein polynomials as smooth weight functions [9]. Esi et al. studied statistical convergence and derived new theorems for Bernstein polynomials [10]. In recent years, $(p,q)$-calculus has been progressed rapidly by many researchers in [11–13], and [14].

In analytic number theory, Bernstein polynomials are an important polynomials family because they have very close relations with special numbers and polynomials such as Stirling numbers, Bernoulli polynomials, Euler polynomials, and so on. Obtaining the generating function for the Bernstein polynomials [15] has led to an increase and acceleration of studies in this area. For instance, Araci and Acikgoz derived some combinatorial relations and properties between Bernstein and Frobenius–Euler polynomials by using the concept of generating function and fermionic *p*-adic integral on $\mathbb{Z} _{p}$ [16]. Acikgoz et al. obtained a new forming between *q*-Bernoulli numbers and polynomials and *q*-Bernstein polynomials (see [17] and [18]). Kim proposed new *q*-Bernstein polynomials and showed some of their representations by using the fermionic *p*-adic integral [19]. Bayad and Kim found some theorems and corollaries related to *q*-Bernoulli, *q*-Euler, and Bernstein polynomials [20]. Simsek et al. gave some new results for Bernstein and *q*-Bernstein polynomials by using some special functions, polynomials, and numbers ([21, 22], and [23]).

In this article, our main goal is to obtain a generating function of $(p,q)$-Bernstein polynomials by using the $(p,q)$-calculus tools as $(p,q)$-derivative operator, $(p,q)$-exponential functions, and $(p,q)$-hypergeometric functions. After that, we present some theorems and identities by using the mathematical operators on a generating function of $(p,q)$-Bernstein polynomials.

## Miscellaneous definitions and notations

In this part, we give definitions and notations related to $(p,q)$-calculus and use some of them in the subsequent chapters.

Let $0< q< p\leq1$ and $n\in \mathbb{Z} $. Then the $(p,q)$-analogue of *n* is defined by

2.1 $$ [ n ] _{p,q}=\frac{p^{n}-q^{n}}{p-q}. $$

Some $(p,q)$-integers are shown as follows:

2.2 $$\begin{aligned} \begin{aligned} &[ 1 ] _{p,q} =1, \\ &[ 2 ] _{p,q} =p+q , \\ &{}[ 3 ] _{p,q} =p^{2}+pq+q^{2}, \\ &\quad \text{and so on,} \end{aligned} \end{aligned}$$

see [24, 25], and [26].

The $(p,q)$-Binomial coefficients are defined as follows:

2.3 $$ \left [ \begin{matrix} n \\ k\end{matrix} \right ] _{p,q}=\frac{ [ n ] _{p,q}!}{ [ k ] _{p,q}! [ n-k ] _{p,q}!}, $$

where

$$ [ n ] _{p,q}!= \textstyle\begin{cases} 1,&n=0, \\ [ n ] _{p,q}\cdot [ n-1 ] _{p,q}\cdots [ 1 ] _{p,q},&n\neq0,\end{cases}\displaystyle \quad\text{(cf. [24, 25], and [26]).} $$

The $(p,q)$-derivative operator is determined as (2.4)

2.4 $$ D_{p,q} \bigl[ f(x) \bigr] =\frac{f(px)-f(qx)}{(p-q)x}, $$

where $f:\mathbb{R} \rightarrow \mathbb{R} $ and $x\in \mathbb{R}$ (cf. [24, 25], and [26]).

We have two types of $(p,q)$-exponential functions as in the *q*-calculus. These functions are defined as follows:

2.5 $$\begin{aligned} \begin{aligned} &e_{p,q}(x) =\sum_{n=0}^{\infty}p^{\binom{n}{2}} \frac{x^{n}}{ ( ( p,q ); ( p,q ) ) _{n}}, \\ &E_{p,q}(x) =\sum_{n=0}^{\infty}q^{\binom{n}{2}} \frac{x^{n}}{ ( ( p,q ) ; ( p,q ) ) _{n}}, \end{aligned} \end{aligned}$$

where

$$\bigl( ( p,q ) ; ( p,q ) \bigr)_{n}:=\prod _{k=1}^{n-1} \bigl(p^{k}-q^{k} \bigr) $$

is called a $(p,q)$-analog of *q*-shifted operator (cf. [24, 25], and [26]). This operator is described by the following identity:

2.6 $$ \bigl( ( 1,a ) ; ( p,q ) \bigr) _{n}= \bigl( ( 1,a ) ; ( p,q ) \bigr) _{k} \bigl( \bigl( p^{k},aq^{k} \bigr) ; ( p,q ) \bigr) _{n-k}. $$

The hypergeometric series concept has moved to $(p,q)$-calculus and was studied in detail by researchers (see [24] and [25]). The $(p,q)$-hypergeometric series are given by the following equation:

2.7 $$\begin{aligned} &{}_{r}\Phi_{s} \bigl( ( a_{1p},a_{1q} ) ,\ldots, ( a_{rp},a_{rq} ), ( b_{1p},b_{1q} ),\ldots, \bigl( ( b_{sp},b_{sq} ) \bigr), ( p,q ),z \bigr) \\ &\quad=\sum_{n=0}^{\infty}\frac{ ( ( a_{1p},a_{1q} ) ,\ldots, ( a_{rp},a_{rq} ), ( p,q ) ) _{n}}{ ( ( p,q ), ( b_{1p},b_{1q} ),\ldots, ( ( b_{sp},b_{sq} ) ), ( p,q ) ) _{n} ( ( p,q ), ( p,q ) ) _{n}} \\ &\qquad{}\times \biggl( ( -1 ) ^{n} \biggl( \frac{q}{p} \biggr) ^{\binom{n}{2}} \biggr) ^{1+s-r}, \end{aligned}$$

where

$$ \bigl( ( a_{1p},a_{1q} ),\ldots, ( a_{rp},a_{rq} ), ( p,q ) \bigr) _{n}=\prod_{j=1}^{r} \bigl( ( a_{1p},a_{1q} ), ( p,q ) \bigr) _{n}. $$

By using the definition of (2.6), we obtain $(p,q)$-Binomial coefficients by means of $(p,q)$-hypergeometric series as follows:

2.8 $$ _{r}\Phi_{s} \bigl( ( a,b ),-, ( p,q ),z \bigr) = \frac{ ( ( p,bz ) ; ( p,q ) ) _{\infty }}{ ( ( p,az ) ; ( p,q ) ) _{\infty}}. $$

Substituting $( a,b ) = ( 1,0 ) $ and $( 0,1 ) $ into (2.7), we have $(p,q)$-exponential functions by aid of $(p,q)$-hypergeometric series in the following theorem:

2.9 $$ e_{p,q}(x)={}_{r}\Phi_{s} \bigl( ( 1,0 ),-, ( p,q ) ,z \bigr) $$

and

2.10 $$ E_{p,q}(x)={}_{r}\Phi_{s} \bigl( ( 0,1 ),-, ( p,q ) ,z \bigr). $$

Now, we give a $(p,q)$-exponential function by using $(p,q)$-factorial in the following identity:

2.11 $$ \varepsilon_{p,q} ( x ) =\sum_{n=0}^{\infty}p^{\binom {n}{2}} \frac{x^{n}}{ [ n ] _{p,q}!}. $$

We have some results between (2.5) and (2.11) as follows:

2.12 $$\begin{aligned} \begin{aligned} &\varepsilon_{p,q} ( x ) =e_{p,q} \bigl( ( p-q ) x \bigr),\quad 0< q< p\leq 1, \\ &\varepsilon_{\frac{q}{p}} ( x ) =E_{p,q} \bigl( ( p-q ) x \bigr),\quad \biggl\vert \frac{q}{p} \biggr\vert < 1. \end{aligned} \end{aligned}$$

### Remark 1

(cf. [27])

*q*- and $(p,q)$-integers are a good bit similar. However, they have an important difference. One may obtain an equality by using the definition of $(p,q)$-integer as follows:

2.13 $$ [ n ] _{p,q}=p^{n-1} [ n ] _{\frac{q}{p}}. $$

If we take $p=1$ in (2.13), $[ n ] _{p,q}$ reduces to $[ n ] _{q}$, but the opposite is not true. That is, we cannot obtain $[ n ] _{p,q}$ by using $[ n ] _{q}$. Therefore, we can describe that *q*-integers are a special case of $(p,q)$-integers.

## Main results

In this section, we start with definition for $(p,q)$-Bernstein polynomials by means of $(p,q)$-analog of *q*-shifted operator. Thanks to this definition, we give the generating function for $(p,q)$-Bernstein polynomials. An important application of this function is a method for representing $(p,q)$-Bernstein polynomials in different ways.

We begin by giving a definition of $(p,q)$-Bernstein polynomials in the following equation.

### Definition 1

(cf. [28])

Let *k* and *n* be arbitrary positive integers. The $(p,q)$-Bernstein polynomial of degree *n* is given by

3.1 $$ B_{k,n}(x;p,q)=p^{\binom{k}{2}-\binom{n}{2}} \left [ \begin{matrix} n \\ k\end{matrix} \right ]_{p,q}x^{k}(1-x)_{p,q}^{n-k}, \quad k\leq n, $$

where $\frac{n(n-1)}{2}$ is shown by $\binom{n}{2}$.

We note that when $p=1$, Definition 1 reduces to the results of Goldman et al. [1] and Oruc and Philips [6].

These polynomials are given as follows:

3.2 $$ B_{k,n}(x;p,q)=\frac{p^{\binom{k}{2}-\binom{n}{2}} ( ( 1,x ) ; ( p,q ) ) _{k} ( ( p^{k},q^{k+1} ) ; ( p,q ) ) _{n-k}x^{k}}{ ( ( p,q ) ; ( p,q ) ) _{n-k}}. $$

### Generating function of $( p,q ) $-Bernstein polynomials

So far we have mentioned $(p,q)$-calculus and Bernstein polynomials. We turn our attention now to the concept of the generating function of these polynomials based on $(p,q)$-calculus.

By using an approach similar to that of Goldman et al. [1], we arrive at the following theorem.

#### Theorem 1

*For* $0< q< p\leq1$,

3.3 $$ \digamma_{k}^{p,q} ( x,t ) =p^{\binom{k}{2}-\binom {n}{2}} \frac{ ( xt ) ^{k}}{ [ k ] _{p,q}!}\frac{\varepsilon _{p,q}(t)}{\varepsilon_{p,q}(xt)}=\sum_{n=k}^{\infty}B_{k,n}(x;p,q) \frac {t^{n}}{ [ n ] _{p,q}!}. $$

#### Proof

The proof of this theorem is similar to those of Goldman et al. [1] and [22]. Let $\digamma_{k}^{p,q} ( x,t ) $ and $\mathbf{F}_{k}^{p,q} ( x,t,c ) $ be generating functions of $(p,q)$-Bernstein polynomials. So we have the following formal definitions, respectively:

3.4 $$ \digamma_{k}^{p,q} ( x,t ) =\sum _{n=k}^{\infty }B_{k,n}(x;p,q)\frac{t^{n}}{ [ n ] _{p,q}!} $$

and

3.5 $$ \mathbf{F}_{k}^{p,q} ( x,t,c ) =\sum _{n=k}^{\infty }B_{k,n}(x;p,q)\frac{t^{n}}{ ( ( 1,c ) ; ( p,q ) ) _{n}}. $$

By substituting the right-hand side of (3.2) into (3.5), we have

3.6 $$\begin{aligned} &\mathbf{F}_{k}^{p,q} ( x,t,c ) \\ &\quad=\sum _{n=k}^{\infty }\frac{p^{\binom{k}{2}-\binom{n}{2}} ( ( 1,x ) ; ( p,q ) ) _{k} ( ( p^{k},q^{k+1} ) ; ( p,q ) ) _{n-k}x^{k}}{ ( ( p,q ) ; ( p,q ) ) _{n-k}}\frac{t^{n}}{ ( ( 1,c ) ; ( p,q ) ) _{n}} \\ &\quad=\frac{x^{k}t^{k}p^{\binom{k}{2}}}{ ( ( 1,c ) ; ( p,q ) ) _{k}}\sum_{n=k}^{\infty} \frac{p^{-\binom{n}{2}} ( ( 1,x ) ; ( p,q ) ) _{k} ( ( p^{k},q^{k+1} ) ; ( p,q ) ) _{n-k}}{ ( ( p,q ) ; ( p,q ) ) _{n-k} ( ( p^{k},cq^{k} ) ; ( p,q ) ) _{n-k}}t^{n-k} \\ &\quad={}_{2}\Phi_{1} \bigl( ( 1,x ), \bigl( p^{k+1},q^{k+1} \bigr) ; \bigl( p^{k},cq^{k} \bigr), ( p,q ),t \bigr) \frac {x^{k}t^{k}p^{\binom{k}{2}-\binom{n}{2}}}{ ( ( 1,c ) ; ( p,q ) ) _{k}}. \end{aligned}$$

From (3.4) and (3.5), we have the following equality:

3.7 $$\begin{aligned} \digamma_{k}^{p,q} ( x,t ) &=\sum _{n=k}^{\infty }B_{k,n}(x;p,q)\frac{t^{n}}{ [ n ] _{p,q}!} \\ &=\sum_{n=k}^{\infty}B_{k,n}(x;p,q) \frac{ ( p-q ) ^{n}t^{n}}{ ( ( p,q ) ; ( p,q ) ) _{n}} \\ &=\mathbf{F}_{k}^{p,q} \bigl( ( 1,x ), ( p-q ) t, ( p,q ) \bigr). \end{aligned}$$

From the right-hand side of (3.7), we get

$$\begin{aligned} &\mathbf{F}_{k}^{p,q} \bigl( ( 1,x ), ( p-q ) t, ( p,q ) \bigr) \\ &\quad=\sum_{n=k}^{\infty}\frac{p^{\binom{k}{2}-\binom{n}{2}} ( ( 1,x ) ; ( p,q ) ) _{k} ( ( p^{k},q^{k+1} ) ; ( p,q ) ) _{n-k}x^{k}}{ ( ( p,q ) ; ( p,q ) ) _{n-k} ( ( p,q ) ; ( p,q ) ) _{n-k}} \frac{ ( p-q ) ^{n}t^{n}}{ ( ( p,q ) ; ( p,q ) ) _{n}} \\ &\quad=\frac{p^{\binom{k}{2}} ( x ( p-q ) t ) ^{k}}{ ( ( p,q ) ; ( p,q ) ) _{k}}\sum_{n=k}^{\infty } \frac{ ( ( 1,x ) ; ( p,q ) ) _{n-k} ( ( p^{k},q^{k+1} ) ; ( p,q ) ) _{n-k}t^{n-k}}{ ( ( p,q ) ; ( p,q ) ) _{n-k} ( ( p^{k+1},q^{k+1} ) ; ( p,q ) ) _{n-k}} \\ &\quad={}_{1}\Phi_{0} \bigl( ( 1,x ),-; ( p,q ), ( p-q ) t \bigr) \frac{p^{\binom{k}{2}-\binom{n}{2}} ( x ( p-q ) t ) ^{k}}{ ( ( p,q ) ; ( p,q ) ) _{k}} \end{aligned}$$

by using the definition of $(p,q)$-hypergeometric functions

$$\begin{aligned} &=\frac{p^{\binom{k}{2}-\binom{n}{2}} ( x ( p-q ) t ) ^{k}}{ ( ( p,q ) ; ( p,q ) ) _{k}}\frac { ( ( p,x ( p-q ) t ), ( p,q ) ) _{\infty}}{ ( ( p, ( p-q ) t ), ( p,q ) ) _{\infty}} \\ &=p^{\binom{k}{2}-\binom{n}{2}}\frac{ ( xt ) ^{k}}{ [ k ] _{p,q}!}\frac{ ( ( p,x ( p-q ) t ), ( p,q ) ) _{\infty}}{ ( ( p, ( p-q ) t ) , ( p,q ) ) _{\infty}} \\ &=p^{\binom{k}{2}-\binom{n}{2}}\frac{ ( xt ) ^{k}}{ [ k ] _{p,q}!}\frac{e_{p,q}( ( p-q ) t)}{e_{p,q}( ( p-q ) xt)}. \end{aligned}$$

From (2.12), we obtain that

$$ \digamma_{k}^{p,q} ( x,t ) =p^{\binom{k}{2}-\binom {n}{2}} \frac{ ( xt ) ^{k}}{ [ k ] _{p,q}!}\frac{\varepsilon _{p,q}(t)}{\varepsilon_{p,q}(xt)}. $$

Therefore, we arrive at the desired result. □

#### Remark 2

The above defined function can be called a generating function of $(p,q)$-Bernstein polynomials because when $p=1$, the generating function reduces to the generating function of *q*-Bernstein polynomial in [1]. However, the $(p,q)$-Bernstein polynomials are related to $\varepsilon _{p,q}(x)$. We say that $\varepsilon_{p,q}(x)$ converges by using the ratio test, and also this function is well defined for all $\vert x \vert <\frac{1}{ \vert 1-\frac{q}{p} \vert }$ if $\vert \frac{q}{p} \vert <1$.

### The graph of a generating function of $( p,q ) $-Bernstein polynomials

**Figure 1:**
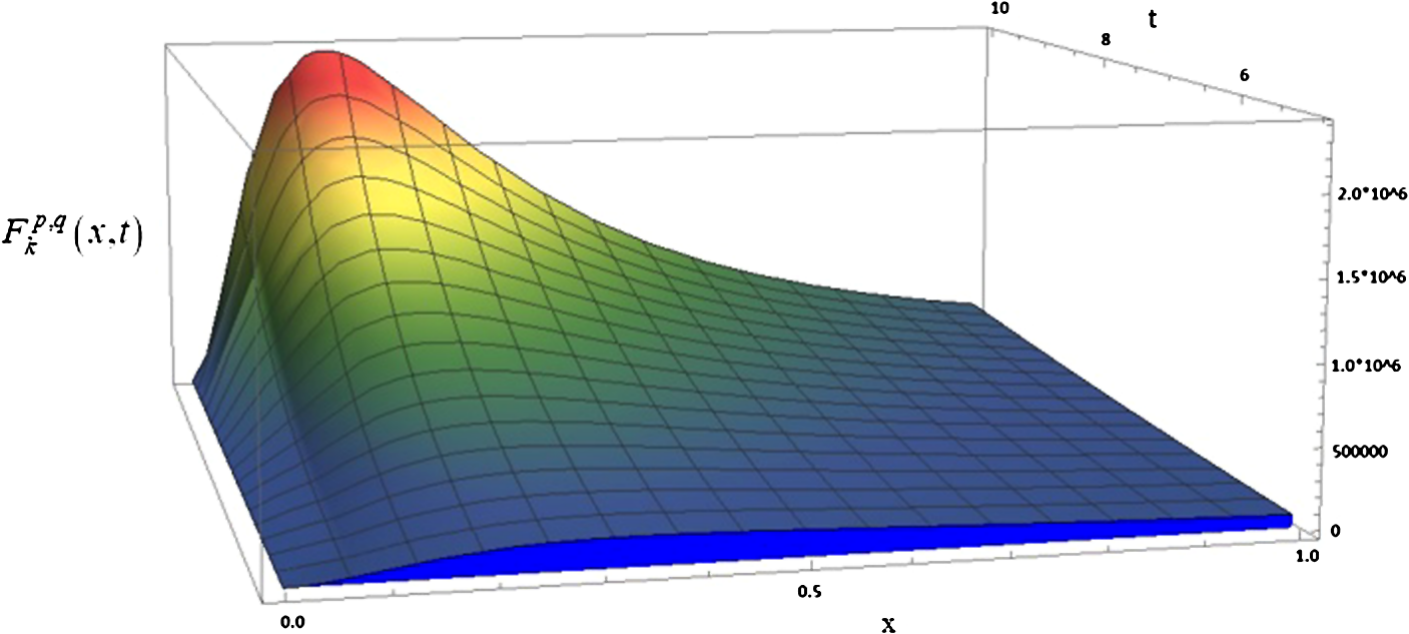
The generating function of $(p,q)$-Bernstein polynomials

In this section, by using a similar method to that of the work of Kucukoglu and Simsek [29], in which a simulation for the extension of unification of Bernstein type basis functions is provided, we provide a simulation for a generating function of the $(p,q)$-Bernstein polynomials with their graph for some special values in Fig. 1.

### Some properties for the generating function of $( p,q ) $-Bernstein polynomials

In this section, by using Theorem 1 in Sect. 3.1, we give some results for the generating function. Having obtained these results, we give some new identities for the $(p,q)$-Bernstein polynomials from arising $(p,q)$-calculus.

Thus we give the following theorem.

#### Theorem 2

*For* $0< q< p\leq1$ *and* $k\in \mathbb{N} \cup \{ 0 \} $,

3.8 $$ \sum_{k=0}^{\infty}\digamma_{k}^{p,q} ( x,t ) z^{k}=\frac {\varepsilon_{p,q}(xtz)\varepsilon_{p,q}(t)}{\varepsilon _{p,q}(xt)}p^{-\binom{n}{2}}. $$

#### Proof

By using a similar method to that of Goldman et al. [1], we can prove this theorem. By using the series expansion for (3.3) depending on *k* and *z*, we have the following equality:

$$\begin{aligned} \sum_{k=0}^{\infty}\digamma_{k}^{p,q} ( x,t ) z^{k} &=\sum_{k=0}^{\infty} \biggl( \frac{ ( xtz ) ^{k}}{ [ k ] _{p,q}!}p^{\binom{k}{2}} \biggr) \frac{\varepsilon _{p,q}(t)}{\varepsilon _{p,q}(xt)}p^{-\binom{n}{2}} \\ &=\frac{\varepsilon_{p,q}(xtz)\varepsilon_{p,q}(t)}{\varepsilon _{p,q}(xt)}p^{-\binom{n}{2}}. \end{aligned}$$

 □

#### Corollary 1

3.9 $$ \sum_{k=0}^{n}B_{k,n}(x;p,q)=1. $$

#### Proof

If we take $z=1$ in (3.8), we obtain the following identity:

3.10 $$ \sum_{k=0}^{\infty}\digamma_{k}^{p,q} ( x,t ) =p^{-\binom {n}{2}}\varepsilon_{p,q}(t). $$

If we expand both sides of (3.3) to a series depending on *k*, we have

$$ \sum_{k=0}^{\infty}\digamma_{k}^{p,q} ( x,t ) =\sum_{k=0}^{\infty } \Biggl( \sum _{n=k}^{\infty}B_{k,n}(x;p,q) \frac{t^{n}}{ [ n ] _{p,q}!} \Biggr). $$

By applying the Cauchy product to the above equation, we get

3.11 $$ \sum_{n=0}^{\infty}\frac{t^{n}}{ [ n ] _{p,q}!}\sum_{k=0}^{n}B_{k,n}(x;p,q)=\sum _{n=0}^{\infty}\frac{t^{n}}{ [ n ] _{p,q}!}. $$

By equating the coefficients of $\frac{t^{n}}{ [ n ] _{p,q}!}$ in (3.11), the desired result is obtained. □

#### Remark 3

When $p=1$, Corollary 1 is reduced to Corollary 4.2 in [1].

#### Corollary 2

$$ \sum_{l=0}^{n-k} \left [ \begin{matrix} n \\ l\end{matrix} \right ]_{p,q}x^{l}p^{\binom{l}{2}}B_{k}^{n-l} ( x;p,q ) =p^{\binom{k}{2}} \left [ \begin{matrix} n \\ k\end{matrix} \right ]_{p,q}x^{k}. $$

#### Proof

We give an alternative form of the generating function for $(p,q)$-Bernstein polynomials as follows:

3.12 $$ \varepsilon_{p,q}(xt)\digamma_{k}^{p,q} ( x,t ) =p^{\binom {k}{2}-\binom{n}{2}}\frac{ ( xt ) ^{k}\varepsilon _{p,q}(t)}{ [ k ] _{p,q}!}. $$

By using the definition of a generating function and Taylor series for $(p,q)$-exponential functions, we obtain

$$ \sum_{n=0}^{\infty}p^{\binom{n}{2}} \frac{ ( xt ) ^{n}}{ [ n ] _{p,q}!}\sum_{n=k}^{\infty}B_{k,n}(x;p,q) \frac{t^{n}}{ [ n ] _{p,q}!}=p^{\binom{k}{2}-\binom{n}{2}}\frac{ ( xt ) ^{k}}{ [ k ] _{p,q}!}\sum _{n=0}^{\infty}p^{\binom{n}{2}}\frac {t^{n}}{ [ n ] _{p,q}!}. $$

By applying the Cauchy product to the above equation, we get

3.13 $$ \sum_{n=0}^{\infty} \left ( \sum _{l=0}^{n-k} \left [ \begin{matrix} n \\ l\end{matrix} \right ]_{p,q}x^{l}p^{\binom{l}{2}}B_{k}^{n-l} ( x;p,q ) \right )\frac {t^{n}}{ [ n ] _{p,q}!}=\sum_{n=0}^{\infty}x^{k}p^{\binom{k}{2}} \left [ \begin{matrix} n \\ k\end{matrix} \right ]_{p,q}\frac{t^{n}}{ [ n ] _{p,q}!}. $$

Comparing the coefficients of $\frac{t^{n}}{ [ n ] _{p,q}!}$ in (3.13), we arrive at the desired result. □

#### Remark 4

When $p=1$, Corollary 2 is reduced to Corollary 4.3 in [1].

### Recurrence relations and derivative of $B_{k,n}(x;p,q)$

In this section, we obtain not only the derivative operator to a generating function of $B_{k,n}(x;p,q)$ but also some relations and properties for $\digamma_{k}^{p,q} ( x,t ) $.

#### Corollary 3

*Let* $0< q< p\leq1$. *Then we have*

3.14 $$ D_{p,q,x} \bigl( \digamma_{k}^{p,q} ( x,t ) \bigr) =p^{\binom{k}{2}-\binom{n}{2}}\frac{t^{k}\varepsilon_{p,q}(t)}{ [ k ] _{p,q}!} \biggl[ ( px ) ^{k} \varepsilon_{p,q}(pxt)+\frac {1}{\varepsilon _{p,q}(qxt)} [ k ] _{p,q}x^{k-1} \biggr], $$

where $\max \{ \vert q/p \vert , \vert x \vert , \vert y \vert \} <1$ and $\vert t \vert <1/ \vert 1-q/p \vert $.

#### Proof

By using the same method as that of Theorem 4.7 in the work of Goldman et al. [1], we can prove this corollary. By using the derivative of the generating function for $B_{k,n}(x;p,q)$ with respect to *x*, we have

$$\begin{aligned} D_{p,q,x} \bigl( \digamma_{k}^{p,q} ( x,t ) \bigr) &= D_{p,q,x} \biggl( p^{\binom{k}{2}-\binom{n}{2}}\frac{ ( xt ) ^{k}}{ [ k ] _{p,q}!} \frac{\varepsilon_{p,q}(t)}{\varepsilon _{p,q}(xt)} \biggr) \\ &= p^{\binom{k}{2}-\binom{n}{2}}\frac{t^{k}\varepsilon _{p,q}(t)}{ [ k ] _{p,q}!}D_{p,q,x} \biggl( \frac{x^{k}}{\varepsilon _{p,q}(xt)} \biggr). \end{aligned}$$

From the division property of $(p,q)$-derivative, we obtain

$$\begin{aligned} D_{p,q,x} \bigl( \digamma_{k}^{p,q} ( x,t ) \bigr) =p^{\binom{k}{2}-\binom{n}{2}}\frac{t^{k}\varepsilon_{p,q}(t)x^{k}}{ [ k ] _{p,q}!} \biggl[ ( px ) ^{k}D_{p,q,x} \biggl( \frac{1}{\varepsilon _{p,q}(xt)} \biggr) + \frac{1}{\varepsilon_{p,q}(qxt)} [ k ] _{p,q}x^{k-1} \biggr]. \end{aligned}$$

After some basic calculations based on $(p,q)$-calculus in the above equation, we arrive at the desired result. □

#### Corollary 4

3.15 $$\begin{aligned} D_{p,q,t} \bigl( \digamma_{k}^{p,q} ( x,t ) \bigr) ={}& \frac {p^{k-1}\varepsilon_{p,q}(pt)x}{\varepsilon_{p,q}(qt)}\digamma _{k-1}^{p,q} ( x,qt ) + \frac{q^{k}\varepsilon_{p,q}(pt)}{\varepsilon_{p,q}(qt)}\digamma_{k}^{p,q} ( x,qt ) \\ &{} - \frac{\varepsilon_{p,q}(xpt)}{\varepsilon_{p,q}(xqt)}\digamma _{k}^{p,q} ( x,qt ). \end{aligned}$$

#### Proof

Multiplying both sides of the generating function for $B_{k,n}(x;p,q)$ in (3.3) by $\varepsilon_{p,q}(xt)$, we have

3.16 $$ \varepsilon_{p,q}(xt)\digamma_{k}^{p,q} ( x,t ) =p^{\binom {k}{2}-\binom{n}{2}}\frac{ ( xt ) ^{k}}{ [ k ] _{p,q}!} \varepsilon_{p,q}(t). $$

By differentiating the equation in (3.16) with respect to *t*, we obtain

3.17 $$\begin{aligned} D_{p,q,t} \bigl( \varepsilon_{p,q}(xt)\digamma_{k}^{p,q} ( x,t ) \bigr) &= D_{p,q,t} \biggl( p^{\binom{k}{2}-\binom{n}{2}}\frac { ( xt ) ^{k}}{ [ k ] _{p,q}!} \varepsilon_{p,q}(t) \biggr) \\ &= p^{\binom{k}{2}-\binom{n}{2}}\frac{x^{k}}{ [ k ] _{p,q}!}D_{p,q,t} \bigl( t^{k}\varepsilon_{p,q}(t) \bigr). \end{aligned}$$

By applying the product rule of $(p,q)$-derivative in (3.17), we get

3.18 $$\begin{aligned} &\digamma_{k}^{p,q} ( x,pt ) \varepsilon _{p,q}(xpt)+\varepsilon _{p,q}(xqt)D_{p,q,t} \bigl( \digamma_{k}^{p,q} ( x,t ) \bigr) \\ &\quad= p^{\binom{k}{2}-\binom{n}{2}}\frac{x^{k}}{ [ k ] _{p,q}!} \bigl( \varepsilon_{p,q}(pt) [ k ] _{p,q}t^{k-1}+ ( qt ) ^{k}\varepsilon_{p,q}(pt) \bigr). \end{aligned}$$

Applying the definition of $\digamma_{k}^{p,q} ( x,t ) $ in (3.18), we complete the proof of the corollary. □

#### Corollary 5

*Let* $0< q< p\leq1$. *Then we have*

3.19 $$\begin{aligned} \digamma_{k}^{p,q} ( xy,t ) =p^{\binom{n}{2}-\binom {s}{2}-\binom{l}{2}} \frac{ [ l ] _{p,q}! [ s ] _{p,q}!}{ [ k ] _{p,q}!}x^{k-l-s}y^{k-s}t^{k-l-s} \digamma_{l}^{p,q} ( x,t ) \digamma_{s}^{p,q} ( x,yt ), \end{aligned}$$

where $\max \{ \vert q/p \vert , \vert x \vert , \vert y \vert \} <1$ and $\vert t \vert <1/ \vert 1-q/p \vert $.

#### Proof

Now, we re-consider $\digamma_{k}^{p,q} ( xy,t ) $ in the following equation:

3.20 $$\begin{aligned} \digamma_{k}^{p,q} ( xy,t ) ={}&p^{\binom{k}{2}-\binom {n}{2}} \frac{ ( xyt ) ^{k}}{ [ k ] _{p,q}!}\frac{\varepsilon _{p,q}(t)}{\varepsilon_{p,q}(xyt)} \\ ={}&p^{\binom{k}{2}-\binom{n}{2}}\frac{ ( xyt ) ^{k}}{ [ k ] _{p,q}!}\frac{\varepsilon_{p,q}(t)}{\varepsilon_{p,q}(xt)}\frac{\varepsilon_{p,q}(xt)}{\varepsilon_{p,q}(yxt)} \\ ={}&p^{\binom{n}{2}-\binom{s}{2}-\binom{l}{2}}\frac{ [ l ] _{p,q}! [ s ] _{p,q}!}{ [ k ] _{p,q}!}x^{k-l-s}y^{k-s}t^{k-l-s} \\ &{}\times \biggl( p^{\binom{l}{2}-\binom{n}{2}}\frac{ ( xt ) ^{l}}{ [ l ] _{p,q}!}\frac{\varepsilon_{p,q}(t)}{\varepsilon _{p,q}(xt)} \biggr) \biggl( p^{\binom{s}{2}-\binom{n}{2}}\frac{ ( xt ) ^{s}}{ [ s ] _{p,q}!}\frac{\varepsilon_{p,q}(t)}{\varepsilon _{p,q}(xt)} \biggr) \\ ={}&p^{\binom{n}{2}-\binom{s}{2}-\binom{l}{2}}\frac{ [ l ] _{p,q}! [ s ] _{p,q}!}{ [ k ] _{p,q}!}x^{k-l-s}y^{k-s}t^{k-l-s}\digamma_{l}^{p,q} ( x,t ) \digamma_{s}^{p,q} ( x,yt ). \end{aligned}$$

From the above equation, we arrive at the desired result. □

#### Corollary 6

3.21 $$ B_{k,n}(xy;p,q)=\sum_{j=0}^{n-k}p^{-\binom{j}{2}}B_{n-j,n}(x;p,q)B_{k,n-j}(y;p,q). $$

#### Proof

By using (3.3) for $k=0$, we obtain the following equality:

3.22 $$\begin{aligned} \digamma_{0}^{p,q} ( x,t ) ={}&\sum _{n=0}^{\infty }B_{0,n}(x;p,q)\frac{t^{n}}{ [ n ] _{p,q}!} \\ ={}&\sum_{n=0}^{\infty}p^{-\binom{n}{2}}\prod _{l=0}^{n-1} \bigl( p^{l}-xq^{l} \bigr) \frac{t^{n}}{ [ n ] _{p,q}!}. \end{aligned}$$

By using (3.3) again in the following equations, we have

3.23 $$ \begin{aligned} &\digamma_{k}^{p,q} ( xy,t ) =\sum _{n=0}^{\infty }B_{k,n}(xy;p,q)\frac{t^{n}}{ [ n ] _{p,q}!}, \\ &\digamma_{k}^{p,q} ( y,xt ) =\sum _{n=0}^{\infty}B_{k,n}(y;p,q)\frac{ ( xt ) ^{n}}{ [ n ] _{p,q}!}. \end{aligned} $$

By substituting (3.22) and (3.23) into (3.19), after some calculations, we arrive at the desired result. □

#### Corollary 7

3.24 $$ p^{-\binom{n}{2}}\prod_{l=0}^{n-1} \bigl( p^{l}-xtq^{l} \bigr) =\sum_{j=0}^{n}p^{- ( \binom{j}{2}+\binom{n-j}{2} ) }B_{j,n}(x;p,q) \prod_{l=0}^{j-1} \bigl( p^{l}-tq^{l} \bigr). $$

#### Proof

Using (3.21) and (3.1) for $k=0$ and $y=t$, we get

3.25 $$ B_{0,n}(xt;p,q)=\sum_{j=0}^{n}p^{-\binom{j}{2}}B_{n-j,n}(x;p,q)B_{0,n-j}(t;p,q) $$

and

$$ B_{0,n}(x;p,q)=p^{-\binom{n}{2}}\prod_{j=0}^{n-1} \bigl( p^{j}-xq^{j} \bigr). $$

After replacing *j* by $n-j$ in (3.25) and some basic calculations, the proof of the corollary is completed. □

### $(p,q)$-analogue of Marsden’s identity

In this section, we construct $( p,q ) $-Marsden’s identity by using $( p,q ) $-Bernstein polynomials.

#### Corollary 8

$$\begin{aligned} &p^{-\binom{n}{2}}\prod_{l=0}^{n-1} \bigl( tp^{l}-xq^{l} \bigr) \\ &\quad=\sum_{j=0}^{n}p^{- ( \binom{j}{2}+\binom{n-j}{2} ) }(-1)^{j}q^{\binom{j}{2}} \frac{B_{j,n}(x;p,q)B_{n-j,n}(t;\frac {p}{q})}{\left [ \begin{matrix} n \\ j\end{matrix} \right ]_{\frac{p}{q}}},\quad \biggl\vert \frac{p}{q} \biggr\vert >1. \end{aligned}$$

#### Proof

By using the same method as that of Corollary 4.15 in Goldman et al. [1] on the interval $[0,1]$, we can prove this corollary. By replacing *t* by $1/t$ in (3.24), we have

3.26 $$ p^{-\binom{n}{2}}\prod_{l=0}^{n-1} \bigl( p^{l}-xt^{-1}q^{l} \bigr) =\sum _{j=0}^{n}p^{- ( \binom{j}{2}+\binom{n-j}{2} ) }B_{j,n}(x;p,q)\prod _{l=0}^{j-1} \bigl( p^{l}-t^{-1}q^{l} \bigr), $$

and then multiplying both sides of (3.26) by $t^{n}$, we get

3.27 $$ p^{-\binom{n}{2}}\prod_{l=0}^{n-1} \bigl( tp^{l}-xq^{l} \bigr) =\sum_{j=0}^{n}p^{- ( \binom{j}{2}+\binom{n-j}{2} ) }B_{j,n}(x;p,q) \prod_{l=0}^{j-1} \bigl( tp^{l}-q^{l} \bigr) t^{n-j}. $$

By using (3.1), we obtain

3.28 $$ t^{n-j}\prod_{l=0}^{j-1} \bigl( tp^{l}-q^{l} \bigr) =(-1)^{j}q^{\binom{j}{2}}t^{n-j} \prod_{l=0}^{j-1} \biggl( 1-t \biggl( \frac{q}{p} \biggr) ^{-l} \biggr) =(-1)^{j}q^{\binom{j}{2}} \frac {B_{n-j,n}(t;\frac{p}{q})}{\bigl[ {\scriptsize\begin{matrix}{} n \cr j\end{matrix}} \bigr]_{\frac{p}{q}}}. $$

By combining (3.27) and (3.28), we derive a $(p,q)$-analogue of Marsden’s identity on the $[ 0,1 ] $. Thus, the proof of the corollary is completed. □

## Conclusion

In this study, we have first introduced a generating function of $(p,q)$ -Bernstein polynomials. By means of this new function, we have constructed many new results and identities which are generalizations of *q*-Bernstein basis polynomials earlier originally introduced by Goldman et al. in [1].

## References

[1] Goldman R., Simeonov P., Simsek Y. (2014). Generating functions for the *q*-Bernstein bases. SIAM J. Discrete Math..

[2] Bernstein S. (1912). Demonstration du théoréme de Weierstrass fondeé sur la calcul des probabilités. Comm. Math. Soc. Charkow..

[3] Ostrovska S. (2003). *q*-Bernstein polynomials and their iterates. J. Approx. Theory.

[4] Ostrovska S. (2009). The convergence of *q*-Bernstein polynomials. Math. Nachr..

[5] Phillips G.M. (1997). Bernstein polynomials based on the *q*-integers. Ann. Numer. Math..

[6] Oruc H., Phillips G.M. (2003). *q*-Bernstein polynomials and Bézier curves. J. Comput. Appl. Math..

[7] Lewanowicz S., Wozny P. (2018). Bezier representation of the constrained dual Bernstein polynomials. Appl. Math. Comput..

[8] Khan K., Lobiyal D.K. (2017). Bézier curves based on Lupaş $(p,q)$-analogue of Bernstein polynomials in CAGD. J. Comput. Appl. Math..

[9] Perez J.M., Palacin A.F. (1987). Estimating the quantile function by Bernstein polynomials. Comput. Stat. Data Anal..

[10] Esi A., Araci S., Acikgoz M. (2016). Statistical convergence of Bernstein operators. Appl. Math. Inf. Sci..

[11] Mursaleen M., Ansari J.A., Khan A. (2015). Some approximation results by $(p,q)$-analogue of Bernstein–Stancu operators. Appl. Math. Comput..

[12] Mursaleen M., Nasiuzzaman N., Nurgali A. (2015). Some approximation results on Bernstein-Schurer operators defined by $(p,q)$-integers. J. Inequal. Appl..

[13] Mursaleen M., Al-Abied A.A.H., Alotaibi A. (2017). On $(p,q)$-Szász–Mirakyan operators and their approximation properties. J. Inequal. Appl..

[14] Kanat K., Sofyalıoglu M. (2018). Some approximation results for Stancu type Lupaş–Schurer operators based on $(p,q)$-integers. Appl. Math. Comput..

[15] Acikgoz M., Araci S. (2010). On the generating function of the Bernstein polynomials. Num. Anal. Appl. Math. Amer. Inst. Phys. Conf. Proc..

[16] Araci S., Acikgoz M. (2012). A note on the Frobenius–Euler numbers and polynomials associated with Bernstein polynomials. Adv. Stud. Contemp. Math..

[17] Acikgoz M., Erdal D., Araci S. (2010). A new approach to *q*-Bernoulli numbers and *q*-Bernoulli polynomials related to *q*-Bernstein polynomials. Adv. Differ. Equ..

[18] Araci S., Acikgoz M. (2015). A note on the values of the weighted *q*-Bernstein polynomials and weighted *q*-Genocchi numbers. Adv. Differ. Equ..

[19] Kim T., Jang L.C., Yi H. (2010). A note on the modified *q*-Bernstein polynomials. Discrete Dyn. Nat. Soc..

[20] Bayad A., Kim T. (2011). Identities involving values of Bernstein, *q*-Bernoulli, and *q*-Euler polynomials. Russ. J. Math. Phys..

[21] Simsek Y., Acikgoz M. (2010). A new generating function of *q*-Bernstein-type polynomials and their interpolation function. Abstr. Appl. Anal..

[22] Simsek Y. (2013). Functional equations from generating functions: a novel approach to deriving identities for the Bernstein basis functions. Fixed Point Theory Appl..

[23] Simsek Y. (2017). On parametrization of the *q*-Bernstein basis functions and their applications. J. Inequal. Spec. Funct..

[24] Chakrabarti R., Jagannathan R. (1991). A $(p,q)$-oscillator realization of two-parameter quantum algebras. J. Phys. A, Math. Gen..

[25] Jagannathan, R., Rao, K.S.: Two-parameter quantum algebras, twin-basic numbers and associated generalized hypergeometric series. arXiv:math/0602613 [math.QA]

[26] Sadjang, P.N.: On the fundamental theorem of $(p,q)$-calculus and some $(p,q)$-Taylor formulas. arXiv:1309.3934 [math.QA]

[27] Gupta V., Aral A. (2016). Bernstein Durrmeyer operators based on two parameters. Facta Univ..

[28] Mursaleen M., Ansari K.J., Khan A. (2015). On $(p,q)$-analogue of Bernstein operators. Appl. Math. Comput..

[29] Kucukoglu I., Simsek Y. (2016). A note on generating functions for the unification of the Bernstein type basis functions. Filomat.

